# The newly discovered glymphatic system: the missing link between physical exercise and brain health?

**DOI:** 10.3389/fnint.2024.1349563

**Published:** 2024-04-16

**Authors:** Raphael Lopes Olegário, Otávio Toledo Nóbrega, Einstein Francisco Camargos

**Affiliations:** ^1^Graduate Program in Medical Sciences, Faculty of Medicine, University of Brasília, Brasília, Brazil; ^2^Department of Clinical Medicine, Geriatric Medicine Centre, Brasília University Hospital, Brasília, Brazil

**Keywords:** glymphatic, exercise, dementia, cerebrospinal fluid, beta-amyloid, neurodegenerative diseases, cognition, non-pharmacological therapies

## Abstract

Dementias are responsible for the most frequent neurodegenerative diseases and the seventh leading cause of death worldwide. As a result, there is a growing effort by the neuroscientific community to understand the physiopathology of neurodegenerative diseases, including how to alleviate the effects of the cognitive decline by means of non-pharmacological therapies (e.g., physical exercise). Studies have shown that exercise can improve aspects of brain health related to cognition. However, there still needs to be more knowledge regarding the mechanisms controlling these relationships, and a newly discovered cleansing system in the brain, named the glymphatic system, can be the missing link in this mechanism. The objective of this paper is to review recent findings regarding the potential impacts of physical exercise on the glymphatic system and its implications for the onset of neurodegenerative diseases. Additionally, considering the close interplay between exercise and sleep quality, we aim to explore how sleep patterns may intersect with exercise-induced effects on glymphatic function, further elucidating the complex relationship between lifestyle factors and brain health.

## 1 Introduction

According to the World Health Organization (2023), dementias are responsible for the greatest increase of neurodegenerative diseases and the seventh leading cause of death among all diseases. Consequently, the neuroscientific community is increasingly focused on comprehending the physiological processes involved in neurodegeneration and exploring strategies, both pharmacological and non-pharmacological, to alleviate associated clinical and behavioral symptoms.

One of the main biomarkers of neurodegenerative diseases is the accumulation of aggregated proteins in the cerebrospinal fluid. For instance, the amyloid cascade hypothesis of Alzheimer's disease (AD) proposes that deposition of amyloid β-protein in the brain is a central event in disease pathology (Karran and De Strooper, 2022). Impaired clearance of these substances favors the accumulation of amyloid β-protein in the cerebral parenchyma and disease progression (Jessen et al., 2015; Natário and Aguiar, 2021).

Recent discoveries in neuroscience have shed light on the physiopathology of neurodegenerative diseases, namely the glymphatic system (as a junction of the terms “glia” and “lymphatic”). This previously unknown brain cleansing system operates as a vital clearance mechanism, efficiently removing metabolic wastes from the interstitial space filled with interstitial fluid between neurons, promoting the maintenance of optimal cellular and synaptic function within the central nervous system. It is now understood that the glymphatic system plays a significant role in managing the influx of a large amount of the subarachnoid cerebrospinal fluid through the brain parenchyma and to eject the brain interstitial fluid so to have it cleared via perivenous pathways (Iliff et al., 2012).

Conventionally, the central nervous system was believed to lack lymphatic vessels for filtering the fluid surrounding parenchymal cells and maintaining the environment necessary for the survival and function of brain cells (Hladky and Barrand, 2014; Louveau et al., 2015). Unlike peripheral organs that rely on the lymphatic system to clear waste products resulting from cellular metabolism, such as toxic protein aggregates released into the interstitial fluid, the glymphatic system operates on the principle that cerebrospinal fluid exhibits directional flow within the central nervous system, facilitating the transport of both intrinsic and extrinsic substances in the brain at speeds surpassing the limits anticipated by simple diffusion (Lohela et al., 2022).

For that, specialized glial cells (astrocytes) carry aquaporin-4 (AQP4), a water-selective plasma membrane channel that which is expressed throughout the central nervous system and concentrated in foot-processes at the blood-brain barrier (Verkman et al., 2017). AQP4 supports the mixing of the cerebrospinal with the interstitial fluid, inducing the solute clearance (Iliff et al., 2012). The convection mechanism that allows the exchange within the glymphatic system is mainly driven by cardiac pulsation (Kiviniemi et al., 2016).

Because of its neurophysiological role and impact on biological systems, it is speculated that the glymphatic system can be related to the occurrence of a variety of neurodegenerative diseases (Zhang et al., 2022). Furthermore, it may also play a role in distributing non-waste compounds such as glucose, lipids, and amino acids (Jessen et al., 2015). From that standpoint, the glymphatic system may be pointed out as a new paradigm for studies in neurodegenerative diseases.

Current literature shows that exercise can improve cognitive function in healthy older adults, indicating that different exercise regimens can produce distinct effects on the brain (Norman et al., 2018), with evidence that a combined protocol of moderate aerobic and resistance training would improve the overall cognitive function. Notably, recent studies have suggested that these cognitive benefits may be linked, in part, to the glymphatic system (He et al., 2017; von Holstein-Rathlou et al., 2018; Natário and Aguiar, 2021). However, while these findings are promising, the comprehensive details of the mechanisms involved in exercise's impact on the glymphatic system and cognitive health in older adults are still the subject of ongoing research.

Based on this, we postulate that exercise holds the potential to enhance the functionality of the glymphatic system. This hypothesis finds its basis in a growing body of evidence highlighting the profound impact of exercise on brain health and cognitive function (Norman et al., 2018). Considering the glymphatic system's integral role in upholding neurological wellbeing, it is reasonable to posit that physical exercise may invigorate the efficiency of this system. Consequently, this hypothesis underscores the imperative need for further investigation to unveil the intricate interplay between exercise and the glymphatic system, thus elucidating the mechanisms that underpin exercise-induced cognitive enhancements and their potential ramifications for neurological health.

In this in-depth review, we examine the research findings concerning the connection between the glymphatic system and neurodegenerative diseases. Additionally, our objective is to shed light on the influence of physical exercise on this intricate system. Through this exploration, our goal is to provide a thorough understanding of how exercise could affect brain health and potentially mitigate neurodegenerative conditions.

## 2 Methods

We conducted the review of the electronic literature databases MEDLINE (via PubMed), Web of Science, and Scientific Electronic Library Online (Scielo) to identify peer-reviewed original research studies published in English between January 2010 and August 2023. Table 1 displays the descriptors and syntax utilized based on terms from the Medical Subject Headings. Regarding the included studies, no restrictions on the chosen research type or methodology were applied.

**Table 1:** Electronic databases search strategy.

**Eletronic databases**	**Search strategy**
MEDLINE (via Pubmed), Web of Science, Scielo	(glymphatic OR “glymphatic system”) AND ((exercise OR “physical exercise”) OR (“physical activity” OR aerobic)) AND (dementia OR Alzheimer) AND (“cerebrospinal fluid” OR “perivascular space”)

The eligibility criteria employed for the selection of studies in our review were thoroughly delineated. Initially, we sought studies that delved into topics regarding the glymphatic system, particularly emphasizing perivascular spaces. Subsequently, our analysis was restricted to studies involving patients diagnosed with probable AD, with the intent of establishing a direct link to our area of interest. Finally, we exclusively included studies that reported outcomes related to physical exercise, aiming to evaluate the glymphatic system's response to physical activity in individuals with AD. These criteria were systematically applied to ensure the relevance and consistency of the evidence considered in our review. Additionally, studies were deemed pertinent if they delved into the dynamics of cerebrospinal fluid and perivascular spaces within the purview of neurological outcomes.

In the following section, we have categorized our findings to structure a detailed discussion on the various aspects of this relationship, offering insights into how different exercise regimens may impact the glymphatic system and, in turn, influence neurological wellbeing. Furthermore, we have explored the potential implications of these exercise-induced glymphatic changes on the prevention and management of AD, shedding light on the promising avenues for future research and clinical applications in the realm of neurology and cognitive health.

## 3 Results

### 3.1 Physical interventions and brain health

Physical exercise as a non-pharmacological therapy is an effective intervention in the prevention and treatment of several brain diseases, most of the accumulated evidence is expressed by meta-analyses (Jia et al., 2019; Liu et al., 2022). Consistent aerobic exercise training may increase or preserve the regional brain volume in areas associated with cognitive decline and changes in cerebral microvasculature function can be a physiological factor that may elicit exercise-enhanced brain function (Hashimoto et al., 2021). Recognizing the nuanced impact of exercise on brain function is paramount; it hinges upon multiple factors, including the type, duration, intensity, and frequency of physical activity, as well as the duration of engagement in such activities. These variables collectively determine the degree to which exercise influences cognitive health and brain function.

A randomized clinical trial (Hoffmann et al., 2016) showed that a moderate-to-high intensity aerobic exercise in mild AD individuals reduces neuropsychiatric symptoms. Moreover, a meta-analysis (Huang et al., 2022) evaluated the impact of various physical exercise interventions on cognitive function among individuals with mild cognitive impairment or dementia, having found that resistance exercise had the highest probability of being the optimal exercise therapy to slow cognitive decline, especially for individuals with probable dementia.

Both human and non-human experimental literature suggest that physical exercise may positively impact the brain function and cognitive performance (to illustrate, see Voss et al., 2013). Although the number of studies on physical exercise is more extensive for older adults than for younger groups, most data suggest that physical exercise can have beneficial effects throughout the lifespan, even for individuals with neurodegenerative diseases (Hillman et al., 2008).

Furthermore, neurotrophic factors, including brain-derived neurotrophic factor, insulin-like growth factor 1, and vascular endothelial growth factor are essential regulators for the effects of physical exercise on brain plasticity during development and adulthood (Baek, 2016). Although some studies conducted on human experimental models show some ambiguity regarding the effects of physical exercise on the stimulation and enhancement of brain-derived neurotrophic factor blood concentration levels (Ribeiro et al., 2021).

### 3.2 Relationship and interaction between physical exercise, glymphatic system and cognition: building bridges

The AD and related dementias have multifactorial characteristics involving many etiopathogenic mechanisms, and several intervention options described in the literature. Among the non-pharmacological therapies recurrently mentioned in the medical literature, physical exercise has been seen as effective for prevention and treatment (De la Rosa et al., 2020), as well as to improve cognitive and physiological decline in some cases (Arciero et al., 2023). However, what mechanisms are involved? Could the glymphatic system be a facilitator?

#### 3.2.1 Exercise, AQP4, and glymphatic system

One assumption is that these improvements can be linked to the AQP4. He et al. (2017) evaluated glymphatic clearance and permeability of the blood–brain barrier in aged mice using a vivo two-photon imaging. The mice performed voluntary wheel running exercise and several outcomes were assessed: water-maze cognition, expression of the astrocytic AQP4, astrocyte and microglial activation, accumulation of amyloid β-protein and synaptic function. The physical exercise accelerated glymphatic clearance and improved astrocytic expression of AQP4, in addition to attenuating amyloid β-protein accumulation and neuroinflammation, protecting mice against synaptic dysfunction and decline in cognition space.

Other studies have shown a (in)direct relationship between exercise and the glymphatic system. In an animal model of AD overexpressing Aβ (APP/PS1) and AQP4 knockout mice that have access to running wheels, Liu Y. et al. (2022) investigated whether the glymphatic clearance of extracellular amyloid β-protein is involved in the beneficial effects of exercise. The early exercise intervention improved AQP4 polarization at the perivascular endfeet of astrocytes in the brains of 5-monthold APP/PS1 mice, subsequently promoting glymphatic transport and reducing brain amyloid β-protein load. Importantly, the improvement of glymphatic function through physical exercise was associated with an increased expression of polarized AQP4. Consequently, exercise had no beneficial effects on AQP4 knockout mice, suggesting that AQP4 may be a critical therapeutic target for the potential use of exercise to delay the onset of AD.

#### 3.2.2 Exercise, blood pressure, and glymphatic system

An alternate hypothesis involves blood pressure regulation. Given the links between conditions like hypertension, blood-brain barrier changes, and cognitive issues, physical exercise could enhance cerebrovascular and cognitive functions through blood pressure control (Di Liegro et al., 2019). During exercise, muscle contractions have been demonstrated in animal models to increase lymphatic flow, enhancing the transport of waste and toxins to lymph nodes and the bloodstream for elimination. Regular exercise not only improves circulation but also supports vital glymphatic system flow, thereby boosting waste removal and optimizing the glymphatic system.

Sustaining an optimal mean arterial pressure is of paramount importance in upholding the hydrostatic pressure gradients between the high-pressure choroid plexi and the low-pressure arachnoid granulations. These gradients are postulated as the primary driving force behind convective flow within the brain, where the cerebrospinal fluid system assumes a central role in maintaining homeostasis within the central nervous system (Wichmann et al., 2022). This delicate equilibrium of pressures and fluid dynamics underscores the indispensable function of mean arterial pressure in underpinning the critical functions of both the brain and the broader central nervous system (Wichmann et al., 2022). Furthermore, it's noteworthy to mention that alterations in mean arterial pressure can influence the brain's fluid dynamics, potentially impacting glymphatic function (He et al., 2017).

The artery wall's pace matches that of the cerebrospinal fluid, implying that arterial wall motion is the primary driving mechanism via a process known as perivascular pumping. Increasing blood pressure has little effect on artery diameter, but it alters the pulsations of the arterial wall, increasing backflow and reducing net flow in the perivascular regions (Mestre et al., 2018).

Previous research has convincingly demonstrated that exercise has the potential to enhance the glymphatic activity in the brain (He et al., 2017). Intriguingly, when mice are in motion (von Holstein-Rathlou et al., 2018), there is an unexpected increase in glymphatic input. This surge in glymphatic activity during physical activity suggests a dynamic relationship between exercise and the glymphatic system. Engaging in aerobic exercise has been demonstrated in animal models to increase heart rate, cerebral pulse pressure, and perfusion flow (He et al., 2017). This elevation in physiological parameters may enhance the flow of brain interstitial fluid, potentially stimulating the glymphatic system. This mechanism, observed in animal studies (von Holstein-Rathlou et al., 2018), could aid in the clearance of Aβ and Tau proteins from the brain. Understanding this intricate interplay between exercise, neurotransmitters, and the glymphatic system offers valuable insights into the potential benefits of physical activity for brain health and neurodegenerative disease prevention, with future research poised to uncover novel approaches to enhance brain waste clearance and promote cognitive wellbeing.

#### 3.2.3 Exercise, sleep, and glymphatic system

Sleep plays a pivotal role in maintaining overall health, and the enduring repercussions of consistently poor sleep quality have been correlated with a range of adverse health consequences (Nelson et al., 2022). Studies indicate that rapid eye movement (REM) sleep, characterized by vivid dreaming, and non-rapid eye movement (NREM) sleep encompass distinct phases with unique physiological characteristics (Carley and Farabi, 2016). Understanding these differentiated sleep phases is crucial as they serve diverse functions in memory consolidation, cognitive processing, and overall mental wellbeing.

While sleep is commonly associated with rest and relaxation, it's intriguing to note that glymphatic activity could undergo an increase during this period. The expansion of the interstitial fluid space seems to drive the sleep-induced enhancement of glymphatic function, suggesting that the restorative effects of sleep may stem from the improved removal of potentially neurotoxic waste products that accumulate in the awake central nervous system (Xie et al., 2013; Reddy and van der Werf, 2020). It is believed that when a person experiences sleep difficulties, glymphatic exchange drops significantly, restricting the outflow of excitatory or inflammatory chemicals from the interstitial space, which can affect metabolic balance. The position of the sleeper's head during sleep may be an essential component in the removal of waste products from the brain (Levendowski et al., 2019), the hypothesized mechanisms underlying the impact of posture on clearance seem to arise from the effects of gravity and the constriction of venous drainage from the carotid veins (Reddy and van der Werf, 2020). Research indicates that head posture in a supine position during sleep can negatively influence the elimination of neurotoxic proteins from the brain, potentially leading to the development of neurodegenerative disorders (Levendowski et al., 2019; Yi et al., 2022).

Furthermore, the vast majority of glymphatic clearance takes place during sleep, with a 90% reduction in glymphatic clearance during wakefulness, emphasizing the pivotal role of sleep in facilitating the removal of waste products and toxins from the brain (Reddy and van der Werf, 2020). The combination of exercise and good sleep hygiene may further enhance glymphatic activity, contributing to the efficient removal of neurotoxic substances from the brain and promoting overall brain health.

Persistent short sleep duration in individuals aged 50 and older was associated with a 30% increased risk of dementia (Sabia et al., 2021). Several epidemiological studies addressing insomnia (and other sleep disorders) as risk factor(s) for dementia have been published in the past 3 years. In a pooled cohort study, an inverted U-shaped association between sleep duration and global cognitive decline was found, indicating cognitive impairment in individuals with insufficient ( ≤ 4 h per night) or excessive (≥10 h per night) sleep duration (Ma et al., 2020).

A recent meta-analysis revealed that exercise improves various sleep outcomes, including more global measures of sleep quality (Kelley and Kelley, 2017). Interventions conducted with middle-aged and older adults reported robust results. In these cases, exercise improved sleep efficiency and duration regardless of the mode and intensity of activity, especially in populations suffering from disease (Dolezal et al., 2017). Admitting that exercise can improve sleep quality and that sleep is a crucial aspect of brain health, we could suppose that this intervention occurred through the glymphatic system.

### 3.3 Future challenges and research limitations

Considerable evidence underscores the influence of physical exercise on brain health, bolstering cognitive function while concurrently diminishing the accumulation of pathological plaques in individuals grappling with AD. Furthermore, it is imperative to acknowledge that the beneficial impacts of exercise transcend the realm of Alzheimer's, reaching out to a broad spectrum of neurological conditions, all the while nurturing overall mental wellbeing (Hoffmann et al., 2016; Morris et al., 2017).

Research on the brain's waste removal system is an attractive alternative because it allows for the bulk removal of hard-to-eliminate proteins without the need for specific transporters (Mestre et al., 2020). Despite evidence from experimental research, it is currently unknown whether a glymphatic system for waste drainage, as described in rodents, exists in the human brain (Benveniste et al., 2019). The major limitations related to this system are the characteristics present in the experimental design. Two significant factors that explain why animal experimentation should be interpreted cautiously in relation to human health are the disparities between animal models of disease and human diseases, as well as species differences in physiology and genetics (Akhtar, 2015).

**Figure 1:**
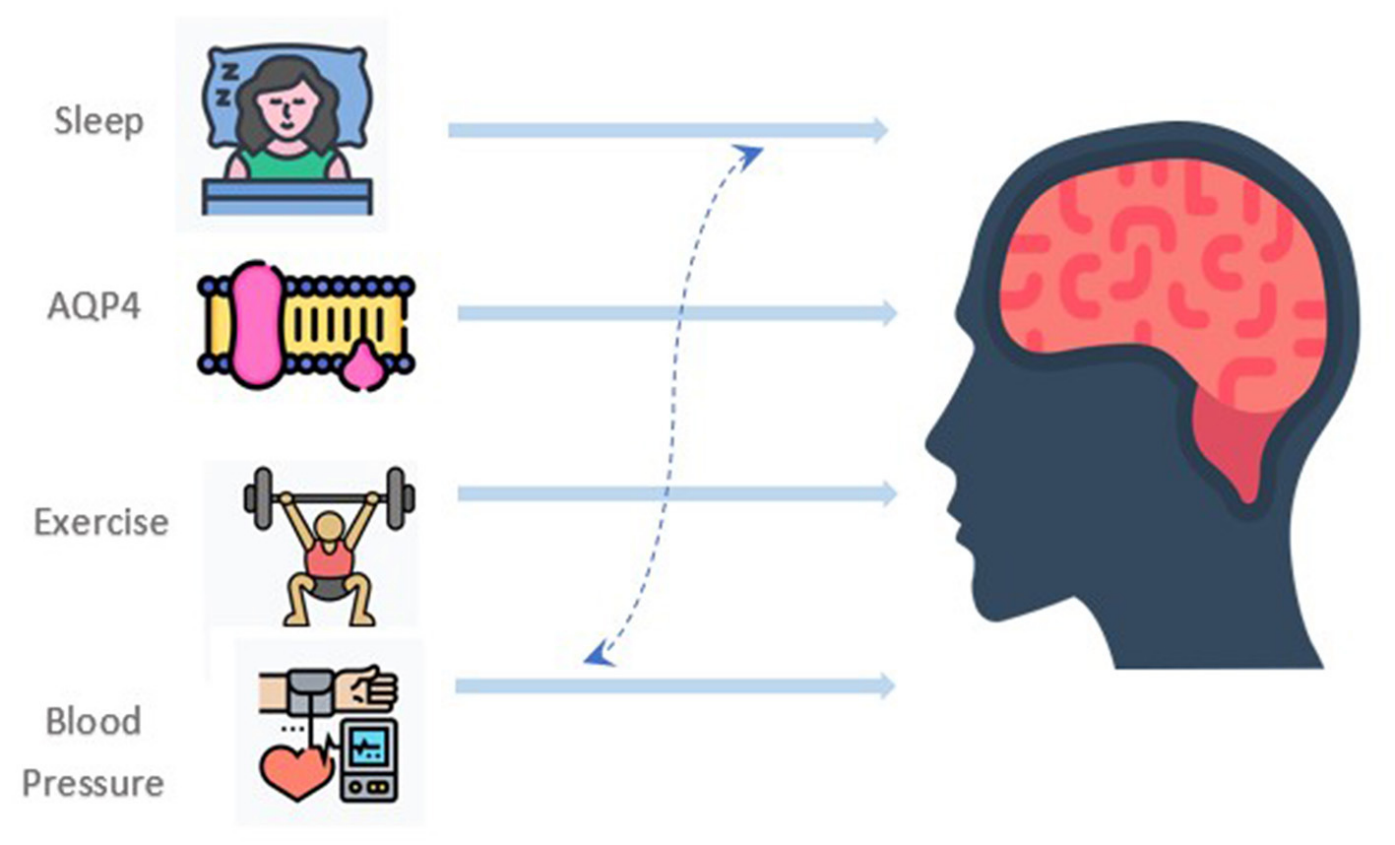
Potential interactions between exercise, sleep quality, blood pressure, and aquaporin-4 (AQP4) in modulating glymphatic system function.

Thus, many issues remain to be addressed. First, is the exercise, to some extent, effective for preventing dementia through glymphatic improvement? What are the types of exercise (strengthening, stretching, balance, or aerobic)? What is the exercise intensity? What is the best period of the day? Is it amelioration based on sleep improvement? Is there any substance intermediating the glymphatic improvement after exercise? And regarding the rule of AQP4? We hope that future research can explore these gaps (Figure 1).

Over the years, our exploration of therapeutic targets for neurodegenerative diseases has been guided by specific pathophysiological hypotheses, encompassing factors such as neuroinflammation, dysregulation of neurotransmitters (e.g., glutamate and acetylcholine), infections, and metal poisoning. Amidst this intricate landscape, the glymphatic system emerges as a novel avenue of inquiry, potentially shedding light on the true benefits of physical exercise for maintaining optimal brain health.

## 4 Conclusion

Our review highlights the burgeoning relationship between the recently uncovered glymphatic system, physical exercise, and their potential impact on neurodegenerative diseases. This exploration underscores the promising link between exercise and brain health via the glymphatic system's role in clearing metabolic waste from the brain. While recognizing the potential of exercise to enhance glymphatic function and cognitive health, further investigations are warranted to decipher the optimal exercise modalities and durations, their impact on sleep quality, and the underlying mechanisms facilitating glymphatic improvement. In our study, it's important to acknowledge a limitation regarding the potential variability in the effectiveness of exercise interventions. We recognize that the efficacy of these interventions may vary depending on factors such as the type, intensity, duration, and frequency of physical activity. This highlights the importance of incorporating a variety of exercises tailored to individual needs and preferences. Despite research limitations, the glymphatic system emerges as a compelling avenue, signaling the potential of exercise not only for immediate cognitive benefits but also as a non-pharmacological strategy to fortify neurological resilience amidst degenerative conditions.

## Author contributions

RLO: Writing – original draft. OTN: Writing – review & editing. EFC: Writing – review & editing.
